# NAVIGATING AGING WHILE CAREGIVING: LONGEVITY PLANNING CONSIDERATIONS FOR CAREGIVERS OF ADULT CHILDREN

**DOI:** 10.1093/geroni/igad104.0176

**Published:** 2023-12-21

**Authors:** Lauren Cerino, Adam Felts, Alexa Balmuth, Julie Miller, Joseph Coughlin

**Affiliations:** Massachusetts Institute of Technology, Cambridge, Massachusetts, United States; Massachusetts Institute of Technology, Cambridge, Massachusetts, United States; Massachusetts Institute of Technology, Cambridge, Massachusetts, United States; AARP, Bethesda, Maryland, United States; Massachusetts Institute of Technology, Cambridge, Massachusetts, United States

## Abstract

Caregiving is a demanding, complex, and costly role. According to AARP (2020), an increasing population of older adults in the U.S. are providing unpaid care to younger adult family members, with over two million adults in the U.S. providing care for an adult child. Caregivers of adult children experience unique challenges that can affect how they plan for their own longevity, as well as their adult child’s longevity. To further explore the experiences of aging caregivers of adult children, the MIT AgeLab conducted an exploratory, qualitative study based on interviews with participants drawn from the MIT AgeLab’s CareHive panel. Through a series of interviews conducted in June 2022, three key transition points in the lives of caregivers of adult children were identified. These transitions included the discovery that the child has special needs, the child’s transition into legal adulthood, and the aging of the caregiver. The last transition point, focused on the aging of the caregiver, often presents complex implications including balancing one’s own care needs with those of their child; navigating difficult family conversations; and preparing for continuation of care after one’s own eventual death. Leveraging insights from these interviews, this session will explore complexities within each of the three key transition points, using Schlossberg’s Transition Theory as a lens for analysis. The session will also identify resources and support desired by caregivers for support in this transition point, shedding a particular light on important considerations for professionals across multiple sectors and industries.

